# Electron spin resonance analysis of photoenzymatic catalysis

**DOI:** 10.1093/nsr/nwae095

**Published:** 2024-03-14

**Authors:** Lu Yu, Changlin Tian

**Affiliations:** High Magnetic Field Laboratory, Hefei Institutes of Physical Science, Chinese Academy of Sciences, China; Division of Life Sciences and Medicine, University of Science and Technology of China, China; High Magnetic Field Laboratory, Hefei Institutes of Physical Science, Chinese Academy of Sciences, China; Division of Life Sciences and Medicine, University of Science and Technology of China, China

## Abstract

This Perspective highlights recent research progress and prospects in elucidating the catalytic mechanism of photoenzymes using ESR (electron spin resonance) spectroscopy, which is emerging as a unique and crucial method for identifying radical intermediates, illustrating electron transfer events and the underlying mechanisms of photoenzymatic catalysis.

The integration of photocatalysis and enzymatic catalysis leads to a cross-disciplinary area, namely photoenzymatic catalysis, which inherits the robust reactivity of photocatalysis and the advantages of enzymatic catalysis, such as mild reaction conditions and high selectivity [1]. Over the past two decades, photoenzymatic catalysis has undergone significant advancements in areas such as asymmetric synthesis and biomanufacturing [2,3]. However, while research efforts have been primarily focused on developing new photoenzymatic reactions, underlying mechanisms of photoenzymes are only sparsely understood. In particular, photoenzymatic catalysis involves highly reactive radical species, singlet states and complicated electron transfer (ET) events between substrate/product, redox enzyme and photosensitizer, requiring sensitive, time-resolved and operando methods to reveal details of catalytic mechanisms and light-driven ET processes (Fig. 1A).

**Figure 1.**
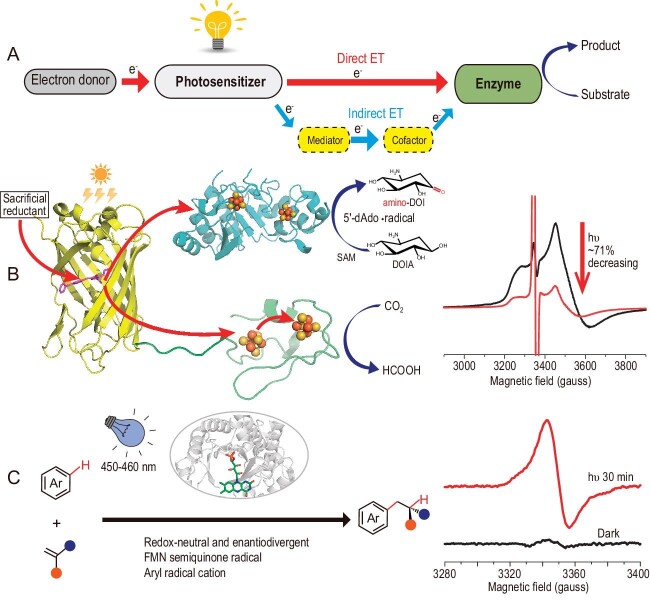
(A) General scheme of electron transfer routes in photoenzymatic catalysis. (B) ESR characterization of photoexcited radical intermediates and ET pathways in PSP2-based photoenzymatic systems. (C) ESR characterization of radical intermediates in flavin-dependent photoenzymatic catalysis.

Electron spin resonance (ESR) spectroscopy is a versatile technique for unraveling catalytic mechanisms involving paramagnetic reactive intermediates, ranging from transition metal ions to free radicals [4]. For open-shell intermediates, ESR spectroscopy provides essential information on electronic structures, spin states and oxidation states, which are fundamental to understanding their reactivity. For photoenzymatic catalysis, which usually involves complex paramagnetic intermediates, ESR spectroscopy is no doubt the technique of choice for gaining insights into the ET routes and catalytic mechanisms. For short-lifetime singlet states and free radicals, ESR sampling with freeze quench and low temperature could extend the window for detection, while the ESR spin trapping technique could characterize radical species with a nanosecond lifetime. Here, we describe some selected examples of studies where ESR spectroscopy was applied to the study of photoenzymatic catalytic processes, in particular for the identification of radical intermediates, and for the illustration of ET events and underlying mechanisms.

Normally, photoenzymatic catalysis is initiated with excitation of photosensitizers, either intrinsic (e.g. flavin mononucleotide (FMN)/flavin adenine dinucleotide (FAD)) or exogenous (e.g. chromophore groups or synthetic rhodium complexes), to promote the electron or energy transfer required to drive the reactions. In our recent work, the reduction of [4Fe-4S] clusters in radical S-adenosylmethionine (SAM) enzymes by photosensitizer protein (PSP2), which was engineered by replacing the chromophore residue Tyr66 in superfolder yellow fluorescent protein (sfYFP) with benzophenone–alanine (BpA), was directly monitored by ESR spectroscopy [5]. The photosensitizer protein could efficiently convert to a long-lived triplet excited state (PSP2*) upon visible-light absorption, and then reacted with NADH to generate a super-reducing radical (PSP2^•^, *E*^0^ = −1.47 V vs. SHE) [6]. A sharp ESR signal at *g* = 2.001 was formed upon light activation, corresponding to the PSP2^•^ radical, while signals with *g* values of 2.04 and 1.91 arose from the reduced [4Fe-4S] cluster (Fig. 1B). Remarkably, ESR spectroscopy unambiguously demonstrated that the PSP2^•^ radical can reduce the auxiliary [4Fe-4S] cluster of BtrN, which has the lowest reduction potential among known radical SAM enzymes and cannot be reduced by dithionite, which suggests new ideas for developing PSP2-based photoenzymes for challenging chemical transformations.

In parallel, a miniature photocatalytic CO_2_-reducing enzyme (mPCE) was designed by fusing PSP2 protein with a ferrodoxin containing two [4Fe-4S] clusters (Fe_A_/Fe_B_) [7]. The light-activated PSP2^•^ radical was proposed to transfer electrons to Fe_A_ and then to Fe_B_, on which CO_2_ was converted into formic acid (FA) (Fig. 1B). In this study, low-temperature and temperature-dependent ESR measurements were applied to not only demonstrate the efficient reduction of Fe_A_/Fe_B_ by the PSP2^•^ radical upon light activation, but also provide valuable information on different dynamic and relaxation properties of Fe_A_ and Fe_B_. Moreover, parallel-mode ESR experiments strongly implied the formation of an all-ferrous [4Fe-4S]^0^ state, which was responsible for reducing CO_2_ to FA through a two-electron process. Combinational studies of ESR measurements, electrochemical experiments and photocatalytic CO_2_ reduction assays, successfully revealed the multi-step photo-induced ET pathway and mechanism of CO_2_ reduction by photoenzyme mPCE.

ESR measurements have also been applied to illustrate the physicochemical properties of radical intermediates in flavin-dependent photoenzymatic catalysis. Recently, we applied ESR spectroscopy to uncover catalytic mechanisms of a new scheme, which catalyzed the non-natural redox-neutral hydroarylation of alkenes with electron-rich arenes [8]. The scheme was based on visible-light excitation of the natural photoenzyme *Cv*FAP and could forge C(sp^2^)–C(sp^3^) bonds in a sustainable and enantiodivergent fashion, offering a novel solution to the challenge of stereochemical control in radical hydroarylation reactions. Using ESR spectroscopy in combination with low-temperature conditions and the spin-trapping technique, the FMN semiquinone radical (FMN_sq_) and the aryl radical cation were unambiguously detected and characterized, supporting the proposed single-electron transfer (SET) oxidation pathway (Fig. 1C). Furthermore, in our recent work, ESR spectroscopic studies elucidated the catalytic mechanism of light-driven enzymatic enantioselective radical acylation [9]. Our collaborators, Prof. X. Huang *et al.* repurposed a thiamine diphosphate (ThDP)-dependent lyase as a stereoselective radical acyl transferase (RAT) through protein engineering and combination with organophotoredox catalysis, achieving high enantioselectivity in preparing diverse chiral ketones from aldehydes and redox-active esters. The ESR method not only detected the formation of a persistent ThDP-derived ketyl radical, but also confirmed the generation of a prochiral benzylic radical. The ESR data clearly demonstrated that enzyme-bound ThDP-derived ketyl radicals are selectively generated through single-electron oxidation by a photoexcited organic dye and then cross-coupled with prochiral alkyl radicals with high enantioselectivity. The relationship between photoexcitation and the evolution of radical

intermediates was monitored by time-dependent ESR spectroscopy. Moreover, ESR experiments under specific conditions showed the essential players to generate these radical intermediates, supporting the proposed dual photoredox and biocatalytic mechanism.

As evidenced above, ESR spectroscopy is extremely valuable in dissecting photoenzymatic mechanisms as it provides detailed information on the radical intermediates and ET events involved. With the development of advanced ESR techniques, unprecedented insights into the roles of radical species, cofactors and metal centers during photoenzymatic reactions will be achieved. For example, high-field/high-frequency ESR could provide improved sensitivity and enhanced resolution for g-factor and fine structure. Moreover, pulsed ESR techniques such as pulsed ENDOR (electron–nuclear double resonance) provide higher resolution as well as allowing observation of weakly coupled nuclei, also offering insights into the relaxation times of the system. Also, TR-ESR (time-resolved ESR) has proven to be a powerful technique for the characterization of the structure and dynamics of transient radicals produced in photoenzymatic reactions. In fact, these advanced ESR techniques have played an essential role in studies of photoenzymes such as photolyases, where pulsed ENDOR unravels the electronic structure and surrounding environment of flavin radical intermediates, while TR-ESR allows a direct tracking of photochemically generated intermediates with a temporal resolution reaching nanoseconds [10]. Furthermore, the integration of ESR spectroscopy with complementary methodologies, such as X-ray crystallography, mass spectrometry and transient absorption spectroscopy, allows for a comprehensive and multifaceted analysis of photoenzymatic catalysis. With increasing attention on green and sustainable production of chemicals and materials using photoenzymes, catalytic mechanisms illustrated by ESR and combinational methods will greatly inspire further development of new photoenzymes with enhanced reactivities and higher enantioselectivities.
